# Factors influencing the prognosis of out-of-hospital cardiac arrest during the coronavirus disease 2019 pandemic: an analysis of the Utstein Registry in Aichi, Japan

**DOI:** 10.1186/s12873-026-01517-8

**Published:** 2026-02-27

**Authors:** Masaki Ito, Toshie Manabe, Tomonori Hattori

**Affiliations:** 1 Department of Advanced Emergency and Disaster Medicine, Nagoya City University Graduate School of Medical Sciences, Nagoya, Japan; 2 College of Nursing, Aichi Medical University, Nagakute, Japan; 3 Nagoya City University Graduate School of Data Science, Nagoya, Japan; 4 Center for Clinical Research, Nagoya City University Hospital, Nagoya, Japan

**Keywords:** Out-of-hospital cardiac arrest, COVID-19 pandemic, Neurological outcome, Utstein Registry, Emergency medical services, Japan

## Abstract

**Background:**

Out-of-hospital cardiac arrest (OHCA) remains a major global public health concern, with survival rates remaining low despite decades of efforts to enhance emergency response systems. The coronavirus disease 2019 (COVID-19) pandemic significantly impacted emergency medical services (EMS), causing longer response intervals, changes in airway management practices, and reduced bystander intervention, which may have altered the prognostic factors associated with OHCA outcomes. This study aimed to identify factors associated with favorable neurological outcomes among patients with OHCA and investigate how these factors changed across three periods: pre-COVID-19 (2017–2019), early COVID-19 (2020–2021), and late COVID-19 (2022–2023).

**Methods:**

We conducted a retrospective observational study using Utstein-style Registry data from Aichi Prefecture, Japan (2017–2023). Patient demographics, bystander interventions, EMS activities, and outcomes were compared across periods. Neurological outcomes were assessed using the Cerebral Performance Category, with scores of 1–2 considered favorable (mortality recorded as Cerebral Performance Category 5). To identify factors independently associated with favorable outcomes, logistic regression analyses were separately performed for each period.

**Results:**

Of 51,890 patients, the proportion of favorable neurological outcomes declined from 4.6% (pre-COVID-19) to 3.4% (early) and 3.1% (late). In the regression model excluding return of spontaneous circulation, the adjusted odds ratio (aOR) for initial ventricular fibrillation/pulseless ventricular tachycardia decreased from 4.340 (pre) to 3.516 (early), whereas the aOR for witnessed arrest increased from 5.866 (pre) to 8.097 (early). During the early period, the association of bystander defibrillation peaked, whereas the negative association of advanced airway management progressively strengthened, with aORs decreasing from 0.268 (pre) to 0.182 (late).

**Conclusions:**

Prognostic factors for favorable neurological outcomes changed across the three COVID-19 periods, indicating that the pandemic altered both the OHCA case mix and the relative importance of key resuscitation elements. Continuous adaptation of EMS systems is crucial for sustaining outcomes under shifting clinical and operational conditions.

## Background

Out-of-hospital cardiac arrest represents a challenging public health issue globally [1]. Despite continued efforts to strengthen emergency medical services (EMS) and promote public awareness, survival rates have only modestly improved in several regions [2, 3]. The “chain of survival,” including early recognition and EMS activation, early bystander cardiopulmonary resuscitation (CPR), prompt defibrillation, advanced life support, and postresuscitation care, remains fundamental to enhancing patient outcomes [4].

Several key factors are associated with OHCA outcomes [5–7]. A previous meta-analysis reported that although epinephrine administration during CPR increased the rate of return of spontaneous circulation (ROSC), patients not receiving epinephrine exhibited higher survival rates with favorable neurological outcomes [8]. Regarding airway management, some studies have suggested that bag–valve–mask ventilation achieves better outcomes than advanced airway management (AAM), whereas others have reported no significant difference [9, 10]. Furthermore, the effects of prehospital AAM seem to vary across various EMS systems [11].

The coronavirus disease 2019 (COVID-19) pandemic, declared by the World Health Organization in March 2020 [12], profoundly impacted emergency medical care worldwide. Previous studies have indicated that in Japan, the proportion of OHCA survivors with favorable neurological outcomes decreased following the pandemic began [13], with similar trends observed in Taiwan [14] and the United States [15]. Prolonged EMS response times, increased AAM implementation [16], and decreased bystander CPR rates in some regions [17] are the factors contributing to this decline. These procedural and behavioral shifts may have altered the determinants of OHCA prognosis.

However, whether the relative importance of each prognostic factor changed over time remains unclear as few studies have directly compared these associations across multiple pandemic phases. Therefore, this study aimed to identify the factors associated with favorable neurological outcomes and investigate how their relative importance changed across three periods (pre-, early, and late COVID-19) using Utstein-style Registry data from Aichi Prefecture, Japan. Understanding these temporal dynamics could provide valuable insights for improving OHCA management during future public health emergencies.

## Methods

### Study design and participants

This retrospective observational study used data obtained from the nationwide Utstein Registry, specifically focusing only on data from Aichi Prefecture, Japan, between January 1, 2017 and December 31, 2023. Aichi Prefecture, located in central Japan, has a population of approximately 7.47 million, making it the fourth most populous region among Japan’s 47 prefectures [18]. The Utstein Registry, a population-based database, collects data on OHCA events using standardized Utstein-style templates [19]. The dataset encompasses data on patient characteristics, bystander interventions, EMS activities, and patient outcomes. The EMS activity records provide data on call receipt time, on-scene arrival time, time to AAM, time to epinephrine administration, and hospital arrival time.

This study included all OHCA cases recorded in the Utstein-style Registry. From this registry, we extracted data on the patients’ characteristics, including age, sex, initial electrocardiogram (ECG) rhythm, location of arrest, and status of witnessed arrest. Data on bystander interventions comprised chest compressions, rescue breathing, and defibrillation. EMS interventions encompassed response time, defibrillation, AAM, intravenous access, and epinephrine administration. Transport time to the hospital was also recorded. Patient outcomes included prehospital ROSC, 1-month survival, and neurological status assessed using the Cerebral Performance Category (CPC) score. Favorable neurological outcome, defined as CPC 1–2 at 1 month, was the primary outcome. Prehospital ROSC and 1-month survival encompassed the secondary outcomes. Moreover, we evaluated whether the associations between key patient/prehospital factors and favorable neurological outcomes varied across the three study periods using period-stratified multivariable models.

### Definition

**Fig. 1:**
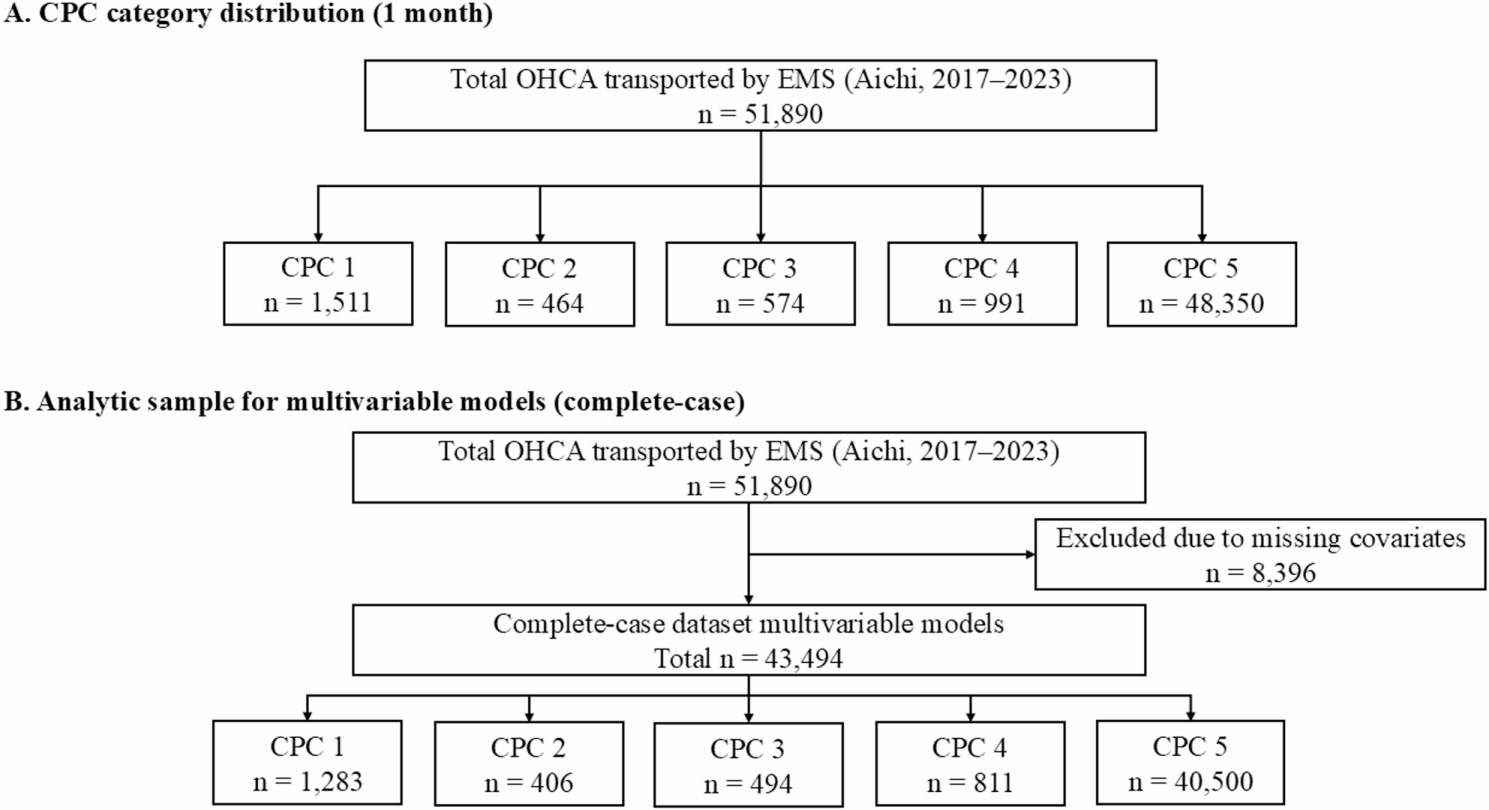
Study flow and outcome hierarchy. Panel **A** showing the Cerebral Performance Category (CPC) scoresamong overall out-of-hospital cardiac arrest (OHCA) casestransported by emergency medical services (EMS). Panel **B** depictingthe analytic sample used for multivariable logistic regression models(complete-case dataset after excluding cases with missingcovariates) and CPCs. In the Utstein Registry, mortality is recordedas CPC 5 and is included in CPC 3–5 (poor outcome)

The observation period in this study was divided into three phases. January 1, 2017–December 31, 2019 was defined as the pre-COVID-19 period, representing a stable, prepandemic baseline. January 1, 2020–December 31, 2021 was defined as the early COVID-19 period, during which repeated states of emergency and behavioral restrictions were implemented. January 1, 2022–December 31, 2023 was defined as the late COVID-19 period, when several public health restrictions had been eased. The CPC scale was employed for assessing neurological outcomes. A CPC score of 1–2 was defined as a favorable neurological outcome (primary outcome), whereas a CPC score of 3–5 was defined as a poor outcome. In the Utstein Registry, mortality is recorded as CPC 5; therefore, all nonsurvivors were included in the poor outcome category (CPC 3–5). In cases with inconsistent 1-month survival status and CPC classification, CPC classification was prioritized for outcome definition, consistent with the Utstein Registry, in which CPC 5 indicates mortality. To improve transparency, we present the CPC category distribution for all OHCA cases transported by EMS, as well as the analytical sample sizes used for the multivariable models in Fig. 1.

Regarding interventions, including chest compressions, defibrillation, AAM, intravenous access, and epinephrine administration, the Utstein dataset included “present” or “absent” records. In the present analysis, following the approach used in previous OHCA registry studies [20], blank entries were treated as “not performed” and were not excluded. This assumption is based on standard EMS reporting practices in Japan, where administered interventions are logged with timestamps.

These temporal cut points were selected a priori to align with major shifts in public health measures and EMS operational constraints while maintaining sufficient sample size and model stability within each period. The study flow diagram summarizing the cohort definition and analytic sample sizes is depicted in Fig. 1.

### Statistical analysis

Categorical variables were summarized as counts and percentages and compared using the chi-square test. Continuous variables were presented as medians with interquartile ranges or means with standard deviations. Following an assessment of normality, comparisons were performed using the Kruskal–Wallis or Mann–Whitney U test, as appropriate.

For regression analyses, to improve interpretability and maintain model parsimony in period-stratified analyses, we dichotomized EMS response time (time from emergency call receipt to EMS arrival at the scene) and transport time (time from EMS arrival at the scene to hospital arrival) at the overall medians (≤ 7 vs. >7 min and ≤ 21 vs. >21 min, respectively).

The patients’ characteristics, resuscitation practices, and outcomes were compared across the three periods. Furthermore, these variables were compared between the groups with favorable and poor neurological outcomes, with subgroup analyses conducted for each period.

Multivariable logistic regression analysis was performed for identifying the factors associated with neurological outcomes, with patient characteristics, bystander interventions, and EMS interventions utilized as independent variables. The covariates were selected on the basis of clinical relevance and statistical significance in the univariable analyses. Logistic regression models were constructed using the forced entry method and separately applied for each period. Interaction terms between covariates were excluded because they were not prespecified and would considerably increase model complexity and risk overfitting with limited interpretability.

Multivariable logistic regression analyses were performed using a complete-case approach; cases with missing covariate data were excluded from the regression models. The following were the final analytic sample sizes for the multivariable models: pre-COVID-19 period, *n* = 16,450; early COVID-19 period, *n* = 12,083; and late COVID-19 period, *n* = 14,961.

All statistical tests were two-tailed, and a p-value of < 0.05 was considered statistically significant. Data analyses were conducted using Statistical Package for the Social Sciences (version 29.0.2, IBM Corp., Armonk, NY, USA). The Utstein-style Registry dataset provided by the Aichi Prefectural Government was accessed for research purposes on March 15, 2025, under data-sharing approval.

## Results

### Temporal trends in clinical characteristics and resuscitation practices

Of 51,890 patients, the numbers of patients with OHCA transported by EMS were 21,455, 14,267, and 16,168 in the pre-, early, and late COVID-19 periods, respectively. The study flow diagram and analytic sample sizes are illustrated in Fig. 1.

The comparison of clinical characteristics and resuscitation practices across the three periods is presented in Table 1. The patients’ age significantly differed among the periods (*p* < 0.001); the patients in the COVID-19 period were generally older than those in the pre-COVID-19 period. Moreover, the distribution of the initial ECG rhythms significantly changed (*p* < 0.001), with the patients in the COVID-19 period demonstrating decreased ventricular fibrillation (VF)/pulseless ventricular tachycardia (VT) rates and increased asystole rates compared with those in the pre-COVID-19 period. The bystander chest compression rates significantly differed across periods (*p* < 0.001), decreasing from 61.8% in the pre-COVID-19 period to 60.0% in the early COVID-19 period. Furthermore, the rates of witnessed arrests significantly declined (*p* < 0.001), with the proportion of witnessed cases decreasing from 42.5% in the pre-COVID-19 period to 40.8% in the early COVID-19 period.

**Table 1:** Temporal trends in the clinical characteristics and resuscitation practices among OHCA patients across three periods

	2017–2019 (*n* = 21,455)	2020–2021 (*n* = 14,267)	2022–2023 (*n* = 16,168)	*p* value
**Demographic**				
Sex, male, n (%)	12,336 (57.5)	8,314 (58.3)	9,203 (56.9)	0.058
Age, median (IQR)	79.0 (69.0–87.0)	80.0 (70.0–87.0)	80.0 (70.0–87.0)	< 0.001
**Patient status**				
Initial rhythm, n (%)				< 0.001
VF/Pulseless VT	1,393 (6.5)	844 (5.9)	946 (5.9)	
PEA	5,298 (24.7)	3,529 (24.7)	3,843 (23.8)	
Asystole	13,698 (63.8)	9,211 (64.6)	10,740 (66.4)	
Others	1,066 (5.0)	683 (4.8)	639 (4.0)	
**Bystander**				
Witnessed, n (%)	9,110 (42.5)	5,815 (40.8)	6,395 (39.6)	< 0.001
Chest compressions, n (%)	13,252 (61.8)	8,554 (60.0)	10,016 (61.9)	< 0.001
**EMS**				
Time from emergency call to EMS arrival, median (IQR), min (*n* = 21,499, 14,265, and 16,165)	7 (6–8)	7 (6–9)	7 (6–9)	< 0.001
Time from EMS arrival to hospital arrival, median (IQR), min (*n* = 21,454, 14,267, and 16,167)	21 (17–25)	21 (17–25)	21 (18–26)	< 0.001
Defibrillation, n (%)	2,088 (9.7)	1,299 (9.1)	1,422 (8.8)	0.006
AAM, n (%)	11,163 (52.0)	8,529 (59.8)	10,341 (64.0)	< 0.001
Epinephrine, n (%)	3,391 (15.8)	2,229 (15.6)	2,540 (15.7)	0.897
**Outcome**				
Prehospital ROSC, n (%)	2,919 (13.6)	1,627 (11.4)	1,715 (10.6)	< 0.001
Survival at 1 month, n (%)	1,689 (7.9)	936 (6.6)	947 (5.9)	< 0.001
CPC 1–2, n (%)	985 (4.6)	488 (3.4)	502 (3.1)	< 0.001

Abbreviations: IQR, interquartile range; VF, ventricular fibrillation; VT, ventricular tachycardia; PEA, pulseless electrical activity; EMS, emergency medical services; AAM, advanced airway management; ROSC, return of spontaneous circulation.; CPC, Cerebral Performance Category; OHCA, out-of-hospital cardiac arrest

Regarding EMS interventions, the proportion of patients receiving AAM significantly increased, from 52.0% in the pre-COVID-19 period to 64.0% in the late COVID-19 period (*p* < 0.001). By contrast, the proportion of patients receiving epinephrine did not significantly differ across the three periods (*p* = 0.897). Moreover, the EMS response time significantly differed across the three periods (*p* < 0.001). Although the median response time remained at 7 min across all periods, the interquartile range widened during the early and late COVID-19 periods (pre, 6–8 min; early, 6–9 min; late, 6–9 min), demonstrating increased variability in response times during the pandemic.

**Fig. 2:**
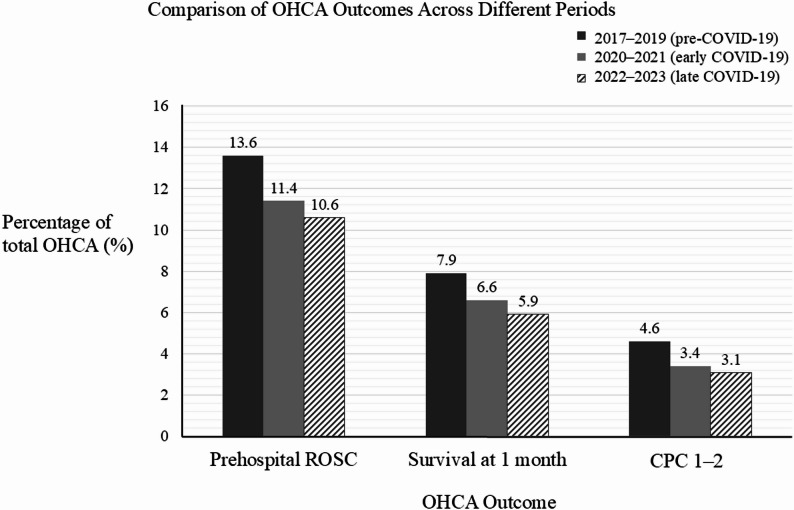
Temporal trends in outcomes among patients with OHCA. The bar chart displaying the changes in the three key outcomes among patients with OHCA: prehospital return of spontaneous circulation (ROSC), 1-month survival, and favorable neurological outcomes (CPC score of 1–2). The y-axis represents the percentage (%) of patients for each outcome. Dark gray bars denote the pre-coronavirus disease 2019 (COVID-19) period (2017–2019), light gray bars denote the early COVID-19 period (2020–2021), and hatched bars denote the late COVID-19 period (2022–2023)

The patient outcomes are shown in Fig. 2. The rates of prehospital ROSC, 1-month survival, and favorable neurological outcomes all significantly decreased across the three periods (*p* < 0.001). The proportion of patients with a CPC score of 1 or 2 diminished from 4.6% in the pre-COVID-19 period to 3.1% in the late COVID-19 period.

### Patients’ demographic and clinical characteristics by neurological outcomes

The comparisons of the patients’ baseline demographic and clinical characteristics by neurological outcomes across the three periods are presented in Table 2. Regarding sex, the favorable outcome group exhibited a significantly higher proportion of males across all periods (*p* < 0.001). The favorable outcome group was significantly younger, with median ages of 67.0, 68.0, and 67.5 years in the pre-, early, and late COVID-19 periods, respectively.

**Table 2:** Demographic and clinical characteristics of OHCA patients by neurological outcomes

	2017–2019 (*n* = 21,455)			2020–2021 (*n* = 14,267)			2022–2023 (*n* = 16,168)		
	CPC 1–2 (*n* = 985)	CPC 3–5 (*n* = 20,470)	*p* value	CPC 1–2 (*n* = 488)	CPC 3–5 (*n* = 13,779)	*p* value	CPC 1–2 (*n* = 502)	CPC 3–5 (*n* = 15,666)	*p* value
**Demographic**									
Sex, male, n (%)	734 (74.5)	11,602 (56.7)	< 0.001	360 (73.8)	7,957 (57.7)	< 0.001	372 (74.1)	8,831 (56.4)	< 0.001
Age, median (IQR)	67.0 (54.0–77.0)	80.0 (69.0–87.0)	< 0.001	68.0 (53.0–79.0)	80.0 (70.0–87.0)	< 0.001	67.5 (53.0–78.0)	81.0 (71.0–87.0)	< 0.001
< 15	24 (2.4)	205 (1.0)	< 0.001	9 (1.8)	114 (0.8)	< 0.001	6 (1.2)	116 (0.7)	< 0.001
15–64	407 (41.3)	3,514 (17.2)		202 (41.4)	2,400 (17.4)		211 (42.0)	2,636 (16.8)	
≥ 65	554 (56.2)	16,751 (81.8)		277 (56.8)	11,265 (81.8)		285 (56.8)	12,914 (82.4)	
**Patient status**									
Initial rhythm, n (%)			< 0.001			< 0.001			< 0.001
VF/Pulseless VT	392 (39.8)	1,001 (4.9)		190 (38.9)	654 (4.7)		222 (44.2)	724 (4.6)	
PEA	226 (22.9)	5,072 (24.8)		104 (21.3)	3,425 (24.9)		91 (18.1)	3,752 (23.9)	
Asystole	35 (3.6)	13,663 (66.7)		22 (4.5)	9,189 (66.7)		21 (4.2)	10,719 (68.4)	
Others	332 (33.7)	734 (3.6)		172 (35.2)	511 (3.7)		168 (33.5)	471 (3.0)	
Onset at home, n (%)	459 (46.6)	13,902 (67.9)	< 0.001	226 (46.3)	9,544 (69.3)	< 0.001	233 (46.4)	10,792 (68.9)	< 0.001
Witnessed, n (%)	830 (84.3)	8,280 (40.4)	< 0.001	423 (86.7)	5,392 (39.1)	< 0.001	427 (85.1)	5,968 (38.1)	< 0.001
Prehospital ROSC, n (%)	829 (84.2)	2,090 (10.2)	< 0.001	400 (82.0)	1,227 (8.9)	< 0.001	424 (84.5)	1,291 (8.2)	< 0.001

Abbreviations: CPC, Cerebral Performance Category; IQR, interquartile range; VF, ventricular fibrillation; VT, ventricular tachycardia; PEA, pulseless electrical activity; ROSC, return of spontaneous circulation

Regarding the initial ECG rhythms, the favorable outcome group demonstrated significantly higher VF/pulseless VT rates across all periods (*p* < 0.001), with the proportion increasing from 39.8% in the pre–COVID-19 period to 44.2% in the late COVID-19 period. Regarding the status of witnessed arrests, > 80% of the patients in the favorable outcome group experienced witnessed arrests, which significantly differed from that of the patients in the poor outcome group (*p* < 0.001), with the proportion increasing from 84.3% in the pre-COVID-19 period to 86.7% in the early COVID-19 period. Prehospital ROSC was achieved in > 80% of the patients in the favorable outcome group across all periods (*p* < 0.001); however, its proportion declined from 84.2% in the pre–COVID-19 period to 82.0% in the early COVID-19 period.

### Bystander and EMS resuscitation practices by neurological outcomes

The comparisons of the bystander and EMS resuscitation practices by neurological outcomes across the three periods are presented in Table 3. In any of the periods, the presence or absence of bystander chest compression was not significantly associated with neurological outcomes. Both bystander and EMS defibrillation were more likely to be performed in the favorable outcome group, which significantly differed from that of the poor outcome group across all periods (*p* < 0.001), with their proportions increasing toward the late COVID-19 period.

**Table 3:** Comparison of bystander and EMS resuscitation practices by neurological outcomes across the three periods

	2017–2019 (*n* = 21,455)			2020–2021 (*n* = 14,267)			2022–2023 (*n* = 16,168)		
	CPC 1–2 (*n* = 985)	CPC 3–5 (*n* = 20,470)	*p* value	CPC 1–2 (*n* = 488)	CPC 3–5 (*n* = 13,779)	*p* value	CPC 1–2 (*n* = 502)	CPC 3–5 (*n* = 15,666)	*p* value
**Bystander**									
Chest compressions, n (%)	559 (60.8)	12,653 (61.8)	0.528	291 (59.6)	8,263 (60.0)	0.881	322 (64.1)	9,694 (61.9)	0.304
	(9.5%) **			(6.7%) **			(3.3%) **		
Rescue breathing, *n* = 16,453, 12,084, and 14,965 (%)	48 (5.9)	631 (4.0)	0.009	20 (4.9)	300 (2.6)	0.004	21 (4.5)	284 (2.0)	< 0.001
Defibrillation, n (%)	144 (14.6)	179 (0.9)	< 0.001	75 (15.4)	121 (0.9)	< 0.001	85 (16.9)	159 (1.0)	< 0.001
	(24.3%) **			(56.9%) **			(87.5%) **		
Time from emergency call to defibrillation by a bystander, *n* = 263, 160, and 197 (%)	3 (1.75–5)	4 (2–6)	0.022	3 (2–5)	4 (2–7)	0.019	4 (1.75–6)	3 (1–6)	0.526
Number of defibrillations, mean (SD) (*n* = 378, 196, and 244)*	1.45 (0.695)	1.39 (0.815)	0.077	1.52 (0.723)	1.64 (1.329)	0.497	1.53 (0.959)	1.81 (1.105)	0.039
**EMS**									
Time from emergency call to EMS arrival, median (IQR), min (*n* = 21,449, 14,265, and 16,165)	7 (5–8)	7 (6–8)	< 0.001	7 (6–9)	7 (6–9)	0.020	7 (6–8)	7 (6–9)	< 0.001
Time from emergency call to EMS arrival ≤ 7 min, *n* = 21,449, 14,265, and 16,165 (%)	638 (64.8)	12,505 (61.1)	0.021	281 (57.6)	7,580 (55.0)	0.263	296 (59.0)	7,934 (50.7)	< 0.001
Defibrillation, n (%)	432 (43.9)	1,656 (8.1)	< 0.001	224 (45.9)	1,075 (7.8)	< 0.001	243 (48.4)	1,179 (7.5)	< 0.001
	(10.7%) **			(10.0%) **			(10.9%) **		
Time from emergency call to defibrillation by EMS, median (IQR), min (*n* = 2,087, 1,299, and 1,421)	9 (7–12)	12 (9–17)	< 0.001	9 (8–13)	12 (9–17)	< 0.001	10 (8–10)	12 (10–19)	< 0.001
Number of defibrillations, mean (SD) (*n* = 2,088, 1,299, and 1,422)*	2.51 (1.854)	2.79 (2.251)	0.327	2.13 (1.618)	2.66 (2.162)	0.006	2.54 (1.984)	2.74 (2.286)	0.694
AAM, n (%)	239 (24.3)	10,924 (53.4)	< 0.001	125 (25.6)	8,404 (61.0)	< 0.001	157 (31.3)	10,184 (65.0)	< 0.001
	(6.7%) **			(5.4%) **			(4.3%) **		
Time from EMS arrival to AAM use, median (IQR), min (*n* = 11,210, 8,528, and 10,338)	7 (5–10)	7 (5–10)	0.965	6 (5–9)	6 (5–9)	0.933	6 (4–8)	6 (5–9)	0.029
Intravenous access, n (%)	228 (23.1)	3,772 (18.4)	< 0.001	92 (18.9)	2,335 (16.9)	0.271	95 (18.9)	2,686 (17.1)	0.299
	(12.0%) **			(10.0%) **			(10.0%) **		
Epinephrine, n (%)	114 (11.6)	3,277 (16.0)	< 0.001	54 (11.1)	2,175 (15.8)	0.005	60 (12.0)	2,480 (15.8)	0.019
	(9.9%) **			(9.3%) **			(10.3%) **		
Time from EMS arrival to epinephrine administration, median (IQR), min (*n* = 3,389, 2,229, and 2,539)	9 (7–12)	9 (8–13)	0.031	8 (7–11.25)	10 (8–15)	0.003	9 (7–10.75)	11 (8–16)	< 0.001
Number of epinephrine administration, mean (SD) (*n* = 3,391, 2,229, and 2,540)*	1.62 (0.963)	2.16 (1.114)	< 0.001	1.48 (0.746)	2.18 (1.150)	< 0.001	1.57 (0.851)	2.21 (1.178)	< 0.001
Time from EMS arrival to hospital arrival, median (IQR), min (*n* = 21,454, 14,267, and 16,167)	21 (17–26)	21 (17–25)	0.581	20 (17–25)	21 (17–25)	0.699	22 (18–27)	21 (18–26)	< 0.001
Time from EMS arrival to hospital arrival ≤ 21 min (*n* = 21,454, 14,267, and 16,167) (%)	532 (54.1)	11,357 (55.5)	0.383	272 (55.7)	7,536 (54.7)	0.648	225 (44.8)	8,166 (52.1)	0.001

Abbreviations: CPC, Cerebral Performance Category; SD, standard deviation; EMS, emergency medical services; IQR, interquartile range; AAM, advanced airway management. * Analyses are restricted to cases with a value of ≥ 1

** Values in parentheses indicate the percentage of missing timestamp data among cases in which the corresponding intervention is performed

The poor outcome group more frequently received EMS-performed AAM, which significantly differed from that of the favorable outcome group across all periods (*p* < 0.001), and its proportion increased from 53.4% in the pre–COVID-19 period to 65.0% in the late COVID-19 period. Similarly, the poor outcome group exhibited a higher proportion of patients receiving epinephrine than the favorable outcome group. The favorable outcome group showed a significantly shorter time from EMS arrival at the scene to epinephrine administration; by contrast, the median administration time in the poor outcome group increased from 9 min in the pre–COVID-19 period to 11 min in the late COVID-19 period. Regarding the time from EMS arrival at the scene to hospital arrival, a significant difference between the favorable and poor outcome groups was noted only in the late COVID-19 period (*p* < 0.001), with the favorable outcome group demonstrating a longer interval.

### Factors influencing neurological outcomes among patients with OHCA

The results of the logistic regression analyses using favorable neurological outcomes (CPC 1–2) as the dependent variable are presented in Tables 4 and 5. The model including ROSC as a covariate is presented in Table 4, whereas the model excluding ROSC is shown in Table 5.

**Table 4:** Multivariable logistic regression of factors for favorable outcomes, including prehospital ROSC

	2017–2019 (*n* = 16,450)				2020–2021 (*n* = 12,083)				2022–2023 (*n* = 14,961)			
	SE	*p* value	Odds ratio	95% CI	SE	*p* value	Odds ratio	95% CI	SE	*p* value	Odds ratio	95% CI
Sex, male	0.106	< 0.001	1.463	1.188–1.801	0.145	0.036	1.354	1.020–1.799	0.140	0.114	1.247	0.948–1.640
Age ≥ 65 years	0.104	< 0.001	0.459	0.374–0.562	0.144	< 0.001	0.443	0.334–0.588	0.135	< 0.001	0.387	0.297–0.504
Initial rhythm: VF/Pulseless VT	0.205	< 0.001	2.749	1.840–4.107	0.294	< 0.001	3.514	1.974–6.257	0.270	< 0.001	3.932	2.314–6.681
Onset at home	0.103	0.251	0.889	0.727–1.087	0.141	0.166	0.822	0.623–1.085	0.136	0.321	0.874	0.670–1.140
Witnessed	0.122	< 0.001	2.709	2.133–3.441	0.175	< 0.001	3.898	2.769–5.489	0.160	< 0.001	2.834	2.071–3.877
Prehospital ROSC	0.114	< 0.001	32.421	25.931–40.535	0.152	< 0.001	32.452	24.068–43.756	0.150	< 0.001	39.355	29.303–52.856
Chest compression by a bystander	0.103	0.098	0.844	0.690–1.032	0.144	0.769	1.043	0.787–1.383	0.140	0.063	1.297	0.986–1.705
Rescue breathing by a bystander	0.228	0.101	0.688	0.440–1.076	0.333	0.415	0.762	0.396–1.465	0.313	0.819	1.075	0.581–1.986
Defibrillation by a bystander	0.211	< 0.001	6.439	4.254–9.746	0.282	< 0.001	5.443	3.135–9.450	0.246	< 0.001	3.064	1.893–4.960
Time from emergency call to EMS arrival ≤ 7 min	0.102	0.505	0.935	0.766–1.140	0.136	0.742	0.956	0.732–1.249	0.129	0.089	1.245	0.967–1.604
Defibrillation by EMS	0.195	< 0.001	2.200	1.502–3.223	0.277	0.047	1.733	1.007–2.985	0.264	0.001	2.345	1.399–3.931
AAM	0.110	< 0.001	0.380	0.306–0.472	0.153	< 0.001	0.263	0.194–0.355	0.149	< 0.001	0.271	0.202–0.362
Intravenous access	0.165	0.114	0.771	0.558–1.064	0.260	0.977	1.007	0.605–1.676	0.262	0.196	0.712	0.426–1.191
Epinephrine	0.188	< 0.001	0.280	0.194–0.404	0.290	< 0.001	0.206	0.117–0.363	0.280	< 0.001	0.281	0.162–0.487
Time from EMS arrival to hospital arrival ≤ 21 min	0.101	0.355	1.098	0.901–1.338	0.139	0.157	1.217	0.927–1.598	0.131	0.177	0.838	0.648–1.083

Abbreviations: SE, standard error; CI, confidence interval; VF, ventricular fibrillation; VT, ventricular tachycardia; ROSC, return of spontaneous circulation; EMS, emergency medical services; AAM, advanced airway management

**Table 5:** Multivariable logistic regression of factors for favorable outcomes, excluding prehospital ROSC

	2017–2019 (*n* = 16,450)				2020–2021 (*n* = 12,083)				2022–2023 (*n* = 14,961)			
	SE	*p* value	Odds ratio	95% CI	SE	*p* value	Odds ratio	95% CI	SE	*p* value	Odds ratio	95% CI
Sex, male	0.093	< 0.001	1.444	1.204–1.731	0.128	0.108	1.228	0.956–1.579	0.122	0.039	1.287	1.013–1.635
Age ≥ 65 years	0.088	< 0.001	0.520	0.437–0.618	0.123	< 0.001	0.531	0.417–0.675	0.115	< 0.001	0.511	0.408–0.640
Initial rhythm: VF/Pulseless VT	0.170	< 0.001	4.340	3.108–6.061	0.250	< 0.001	3.516	2.154–5.738	0.225	< 0.001	4.530	2.912–7.046
Onset at home	0.089	0.017	0.809	0.680–0.962	0.123	0.033	0.769	0.605–0.979	0.117	0.013	0.748	0.595–0.940
Witnessed	0.108	< 0.001	5.866	4.744–7.254	0.159	< 0.001	8.097	5.933–11.048	0.143	< 0.001	6.585	4.977–8.713
Chest compression by a bystander	0.089	0.672	1.039	0.872–1.237	0.124	0.503	1.087	0.852–1.385	0.119	0.006	1.385	1.097–1.748
Rescue breathing by a bystander	0.198	0.256	0.798	0.541–1.178	0.298	0.330	0.748	0.417–1.342	0.281	0.262	1.371	0.790–2.379
Defibrillation by a bystander	0.172	< 0.001	9.534	6.805–13.358	0.228	< 0.001	11.565	7.398–18.079	0.207	< 0.001	5.358	3.574–8.034
Time from emergency call to EMS arrival ≤ 7 min	0.088	0.134	1.141	0.960–1.355	0.118	0.693	1.048	0.832–1.320	0.111	0.001	1.426	1.147–1.774
Defibrillation by EMS	0.164	< 0.001	2.158	1.566–2.974	0.237	< 0.001	2.350	1.477–3.740	0.221	< 0.001	2.924	1.896–4.510
AAM	0.097	< 0.001	0.268	0.222–0.325	0.135	< 0.001	0.211	0.161–0.275	0.128	< 0.001	0.182	0.142–0.234
Intravenous access	0.148	< 0.001	2.070	1.549–2.766	0.243	< 0.001	3.416	2.123–5.496	0.230	< 0.001	2.411	1.535–3.788
Epinephrine	0.178	< 0.001	0.192	0.135–0.272	0.281	< 0.001	0.121	0.070–0.210	0.258	< 0.001	0.178	0.107–0.294
Time from EMS arrival to hospital arrival ≤ 21 min	0.087	0.228	0.901	0.760–1.068	0.120	0.600	0.939	0.743–1.187	0.112	0.002	0.702	0.563–0.875

Abbreviations: SE, standard error; CI, confidence interval; VF, ventricular fibrillation; VT, ventricular tachycardia; EMS, emergency medical services; AAM, advanced airway management

In the model including ROSC, several clinical characteristics were consistently associated with favorable neurological outcomes across all periods. An initial rhythm of VF or pulseless VT, witnessed cardiac arrest, and prehospital ROSC were significantly associated with favorable neurological outcomes across all three periods. Male sex was positively associated with favorable outcomes only in the pre–COVID-19 and early COVID-19 periods, whereas age ≥ 65 years was negatively associated with favorable outcomes across all periods. Regarding resuscitation practices, both bystander and EMS-performed defibrillation were significantly associated with favorable outcomes across all periods. By contrast, EMS-performed AAM and epinephrine administration were negatively associated with favorable neurological outcomes across all periods.

In the model excluding ROSC, several differences emerged compared with the ROSC-adjusted model. Among clinical characteristics, male sex remained significantly associated with favorable outcomes in the pre–COVID-19 and late COVID-19 periods. Cardiac arrest occurring at home was negatively associated with favorable neurological outcomes across all periods. Regarding resuscitation practices, intravenous line establishment was significantly associated with favorable outcomes across all periods; however, the association disappeared when ROSC was included in the model. Additionally, several factors were associated with favorable outcomes only in the late COVID-19 period: bystander-performed chest compressions and shorter EMS response time to scene arrival revealed positive associations, whereas shorter transport time from the scene to hospital arrival exhibited a negative association.

The adjusted odds ratios of several predictors differed across all periods in the model excluding ROSC. The odds ratio for an initial shockable rhythm decreased from the pre–COVID-19 period to the early COVID-19 period. By contrast, the odds ratio for witnessed cardiac arrest increased from the pre–COVID-19 period to the early COVID-19 period. The odds ratios for bystander defibrillation and intravenous access were the highest during the early COVID-19 period. The odds ratio for EMS-performed AAM progressively decreased from the pre–COVID-19 period to the late COVID-19 period.

## Discussion

The main finding of this study is that the prognostic factors associated with favorable neurological outcomes in patients with OHCA shifted across the pre-, early, and late COVID-19 periods. Although key predictors, including shockable rhythm, witnessed arrest, and defibrillation, remained crucial, their relative contributions substantially changed over time. For example, the adjusted odds ratio for an initial shockable rhythm decreased from the pre-COVID-19 period to the early COVID-19 period (4.340–3.516), whereas the association of witnessed arrest strengthened (5.866–8.097). The impact of bystander defibrillation peaked in the early period, whereas the negative association of AAM became stronger in the early COVID-19 period and remained strong in the late COVID-19 period.

Here, witnessed cardiac arrest and bystander-performed defibrillation exhibited particularly high odds ratios as favorable neurological outcome predictors. Their contributions appeared to increase, especially during the early COVID-19 period. During this period, restrictions on movement and stay-at-home orders reduced the incidence of cardiac arrests occurring in public spaces; our findings also demonstrated a reduction in witnessed arrests. Under such circumstances, witnessed events may have become relatively more valuable in determining patient outcomes, potentially explaining the increase in their odds ratios. Similarly, the reductions in witnessed arrests and bystander CPR may have amplified the relative impact of bystander defibrillation on survival. Previous studies have also reported a decline in the witnessed OHCA during the pandemic, supporting the present findings [21–23].

Although an initial shockable rhythm was strongly associated with favorable outcomes, its odds ratio decreased from the pre-COVID-19 period to the early COVID-19 period. The decrease in witnessed arrests during the early COVID-19 period may have partially reduced the prognostic advantage frequently associated with shockable rhythms. In other words, although a shockable rhythm is generally a robust favorable outcome predictor, its benefit may not have been fully realized during the early COVID-19 period owing to increases in unwitnessed arrests and delays in CPR initiation. Conversely, the odds ratio for shockable rhythms increased again in the late COVID-19 period. In this study, the late period was characterized by an older patient population and a higher proportion of asystole. Older age was associated with a lower probability of presenting with a shockable rhythm and poorer survival [24, 25]. Moreover, noncardiac causes of arrest, including respiratory failure and thrombotic complications, reportedly increased during the pandemic [26, 27]. Moreover, our findings suggest that the number of cases requiring longer EMS response times increased from the pre-COVID-19 period to the early COVID-19 period. Delayed EMS arrival may have enabled initially shockable rhythms to deteriorate into asystole before EMS arrival. These changes may have contributed to an increased proportion of nonshockable rhythms and an overall worsening of outcomes. In such a context, the prognostic advantage of shockable rhythms may have become relatively more pronounced, contributing to the increased odds ratio observed in the late COVID-19 period. During the early COVID-19 period, male sex was not significantly associated with favorable neurological outcomes. Previous studies have indicated that males are more likely to present with an initial shockable rhythm than females [28]. However, in the present study, the proportion of VF or pulseless VT decreased during the early pandemic. This shift in rhythm distribution may have reduced the extent to which male sex contributed to favorable outcomes in that period.

Although a positive association was observed between intravenous access and favorable neurological outcomes, this association was not significant in the ROSC-adjusted model. This finding suggests that intravenous access cannot directly enhance neurological outcomes; however, patients who are more likely to achieve ROSC, including those in a relatively better clinical condition, exhibit a higher likelihood of undergoing intravenous access.

Across all periods, AAM was negatively associated with favorable outcomes, with its odds ratio decreasing further in the late COVID-19 period. This finding aligns with previous studies [29] and may reflect the phenomenon known as “resuscitation time bias,” wherein patients with extended arrest duration are more likely to receive AAM [30, 31]. In Japan, guidelines during the COVID-19 pandemic recommended the early use of advanced airway devices for patients with OHCA to mitigate infection risk [32]. Consequently, during the pandemic, the number of AAM procedures increased in our study as well. The guidelines issued by the Japanese Society for Emergency Medicine during the COVID-19 pandemic indicate that chest compressions should be temporarily interrupted to initiate advanced airway insertion and resumed only after confirming successful insertion of an airway device and connection to a bag–valve mask [32]. These interruptions may have adversely affected CPR quality and contributed to the decreasing odds ratio associated with AAM. However, as the registry does not contain data on compression pauses or compression quality, this hypothesis cannot be directly examined.

Only in the late COVID-19 period were bystander chest compression and shorter EMS response time to the scene positively associated with favorable outcomes, whereas shorter transport time to hospital arrival was negatively associated with outcomes. The late period was characterized by deteriorating case mix, including increasing age and a higher proportion of asystole, causing a more limited subset of patients with the potential for survival. In such a context, early stage resuscitation actions, including bystander chest compression and rapid EMS arrival, may have become relatively more crucial. Consequently, factors that were not significant predictors in earlier periods became independent neurological outcome predictors in the late COVID-19 period.

In our multivariable analysis, bystander chest compression was not significantly associated with favorable neurological outcomes in the pre-COVID-19 or early COVID-19 periods. This lack of a statistically significant association should not be interpreted as an evidence of ineffectiveness; rather, it suggests a dilution effect caused by including a large proportion of unwitnessed arrests and those with a noncardiac etiology, wherein the physiological benefit of CPR is inherently limited. Witnessed cardiac-origin arrests demonstrated the most pronounced efficacy of bystander CPR [33], and its impact becomes less apparent when unwitnessed or noncardiac etiologies predominate. Furthermore, heightened public concern about infection transmission during the pandemic may have affected bystanders’ willingness to perform close-contact interventions or altered CPR quality. Considering that the Utstein Registry documents only the presence or absence of CPR, not its quality, such heterogeneity may have further diluted the observed effect. Moreover, powerful covariates, including the initial ECG rhythm, may have overshadowed the independent contribution of CPR in the models.

The observed reduction in favorable neurological outcomes following the start of the COVID-19 pandemic aligns with previous studies [13, 16, 34–36]. The variation in odds ratios and associations of prognostic factors across the periods potentially contributed to this overall deterioration. Specifically, pandemic-related factors, including fewer witnessed arrests due to movement restrictions, shifts in arrest etiology and initial ECG rhythms (possibly associated with delayed EMS response times), and procedural changes in prehospital interventions (e.g., AAM), may have collectively contributed to worsened outcomes. Nevertheless, as an observational study, this analysis cannot establish definitive causal relationships.

### Limitations

The present study had several limitations. First, as a retrospective analysis of data from a single prefecture, the generalizability of the findings might be restricted owing to regional variations in EMS systems and population characteristics. Second, although the study period was divided into the pre-, early, and late COVID-19 periods, the early COVID-19 period encompassed multiple waves of infection and evolving public health measures; thus, this phase could encompass heterogeneous conditions. Third, classifying blank registry entries as “not performed” might have introduced misclassification bias. Fourth, multivariable analyses employed a complete-case approach; cases with missing covariates were excluded from the regression models, and the proportion of excluded cases varied across study periods (Fig. 1), which may have introduced selection bias. Fifth, the dichotomization of EMS response and transport times and the lack of formal interaction testing might have reduced information and restricted inference regarding effect modification across periods; therefore, cross-period differences in adjusted odds ratios should be interpreted descriptively. Finally, residual confounding, including unmeasured factors (e.g., the quality of chest compressions, detailed timing of prehospital interventions, and in-hospital/postresuscitation management), could not be excluded; therefore, prehospital factors alone might not fully explain pandemic-related trends in neurological outcomes.

## Conclusions

This study revealed that the prognostic factors associated with favorable neurological outcomes in patients with OHCA varied across the pre-, early, and late COVID-19 periods. Although key predictors, including shockable rhythm, witnessed arrest, and defibrillation, remained crucial, their relative contributions demonstrated inconsistent magnitudes across periods. The adjusted odds ratio for an initial shockable rhythm decreased from the pre-COVID-19 period to the early COVID-19 period (from 4.340 to 3.516), whereas the association of witnessed arrest strengthened (from 5.866 to 8.097). The impact of bystander defibrillation peaked in the early period, whereas the negative association of AAM became stronger in the early COVID-19 period and remained strong in the late COVID-19 period. These temporal shifts reflect concurrent changes in case mix, arrest circumstances, and emergency medical practices during the pandemic. Understanding how prognostic factors evolve during public health emergencies is significant for strengthening EMS preparedness and optimizing resuscitation strategies in future crises.

## Data Availability

The dataset analyzed during the current study is available from the Aichi Prefectural Government upon reasonable request and with permission from the corresponding author.

## Abbreviations

OHCA: Out-of-hospital cardiac arrest
COVID-19: Coronavirus disease 2019
EMS: Emergency medical services
CPR: Cardiopulmonary resuscitation
ROSC: Return of spontaneous circulation
AAM: Advanced airway management
CPC: Cerebral performance category
ECG: Electrocardiogram
VF: Ventricular fibrillation
VT: Ventricular tachycardia
PEA: Pulseless electrical activity
IQR: Interquartile range
SD: Standard deviation
CI: Confidence interval
